# How a Nutritional Deficiency Became Treated with Fluoride

**DOI:** 10.3390/nu13124361

**Published:** 2021-12-03

**Authors:** Philippe P. Hujoel

**Affiliations:** 1 Department of Epidemiology, School of Public Health, University of Washington, Seattle, WA 98105, USA; hujoel@uw.edu; 2 Department of Oral Health Sciences, School of Dentistry, University of Washington, Seattle, WA 98105, USA

**Keywords:** vitamin D, dental caries, nutritional deficiencies, professional organizations

## Abstract

Ignoring evidence on causes of disease such as smoking can harm public health. This report explores how public health experts started to ignore evidence that pediatric vitamin D deficiencies are associated with dental caries. Historical analyses show that an organization of clinical specialists, the American Dental Association (ADA), initiated this view. The ADA was a world-leading organization and its governing bodies worked through political channels to make fluoride a global standard of care for a disease which at the time was viewed as an indicator of vitamin D deficiencies. The ADA scientific council was enlisted in this endeavor and authorized the statement saying that “claims for vitamin D as a factor in tooth decay are not acceptable”. This statement was ghost-written, the opposite of what the ADA scientific council had endorsed for 15 years, and the opposite of what the National Academy of Sciences concluded. Internal ADA documents are informative on the origin of this scientific conundrum; the ADA scientific council had ignored their scientific rules and was assisting ADA governing bodies in conflicts with the medical profession on advertising policies. The evidence presented here suggests that professional organizations of clinical specialists have the power to create standards of care which ignore key evidence and consequently can harm public health.

## 1. Introduction

Systematic reviews of controlled clinical trials suggest that dental cavities can be prevented with vitamin D supplementation, not with oral hygiene [1,2]. The public health recommendations of some organizations used to be consistent with this evidence. The National Academy of Sciences reported in 1952 that vitamin D supplementation prevented and arrested dental caries [3]. The World Health Organization (WHO) reported in 1984 that self-performed oral hygiene was ineffective to prevent dental caries [4].

Then, these organizations reversed their public health messages in 1989 and 2020, respectively.

The National Academy of Sciences reversed their recommendation in 1989 and described the dental caries prevention claim for vitamin D as unresolved [5]. The WHO reversed their recommendation in 2020 and stated that the “lack of removal of plaque by toothbrushing” can lead to dental caries, contradicting what they reported in 1984 [6]. The clinical trial evidence did not change in the time between these contradictory statements, raising the question as to why these organizations changed their recommendations.

Reversals which are in the opposite direction of clinical trial evidence can cause harm and thus deserve scrutiny. The reversal on the role of a vitamin D deficiency in dental disease etiology was opposite of clinical trial evidence and may still cause harm. Fluoride, regardless of its effectiveness in preventing dental caries, does not prevent the increased chronic disease mortality associated with vitamin D deficiency [7,8].

This report shows that a professional organization of clinical specialists—the American Dental Association (ADA)—was first to reject the evidence that vitamin D deficiencies are a potential cause for dental caries. Other organizations, sometimes decades later, followed by ignoring the vitamin D evidence. The report chronicles the events in four sections: (1) ADA scientific rules and an inexplicable opinion on vitamin D dental caries prophylaxis. (2) How a conflict on advertising policies is informative on the inexplicable, (3) Pathways through which professional organizations can globalize clinical guidelines which ignore or dismiss a preponderance of evidence. And, (4) how the medical management of dental diseases became a historical artefact.

### 1.1. An Inexplicable Opinion

*You cannot sit here and say, “In my judgment”.*

Paul Leech of the American Medical Association instructing the ADA scientific council on the need to adhere to the scientific rules p. 28 in [9].

#### 1.1.1. The ADA as a Trailblazer on Vitamin D Endorsement

Professional organizations of clinical specialists typically derive their scientific gravitas from a scientific council which operates under a set of official rules [10]. At the American Dental Association (ADA) the Council on Dental Therapeutics (ADA CDT) started working in 1930 to fulfill this role. The ADA CDT had adopted a set of official rules from the Council on Pharmacy and Chemistry of the AMA [11]. The ADA CDT was informed that this set of rules should lead to reproducible results; ‘A product (and thus a therapeutic claim for that product) “should be passed on the basis of these rules” [9]. These official rules included a set of scientific criteria aimed to objectively guide the ADA CDT decisions on the authorization of allowable dental health claims. These rules were published in each edition of Accepted Dental Remedies—a yearly publication summarizing the work of the ADA CDT.

The ADA CDT became described in the Journal of the American Dental Association (JADA) as consisting of ‘twelve carefully chosen scientists’ conducting “the unbiased and searching investigation”, and as filling “a definite need in the scientific affairs” of the ADA [12,13].

In August 1930, the ADA CDT endorsed vitamin-D-containing cod liver oil as an “aid in the prophylaxis against caries”. This transformed vitamin D into an accepted dental remedy which could continue to be advertised in JADA [14]. Over 170 such vitamin D advertisements were published in JADA between 1930 and 1945 and approximately one third of them were endorsed by the ADA Seal of Acceptance. Readers of JADA were informed that the ADA Seal implied “acceptance of preparations for therapeutic use” [15].

The ADA CDT authorized the publication of an expert report in support of their endorsement of vitamin D dental caries prophylaxis in 1932 [16]. This expert report cited two comparative trials on cod liver oil published in 1926 (*n* = 32) and 1928 (*n* = 78) [17,18]. A systematic scan of comparative trials indicates that these two trials may have been the only trials accessible to the ADA CDT prior to their endorsement [2]. Whether a third trial published in the British Dental Journal in the early months of 1930 was accessible to the ADA CDT at the time of their decision making is unclear [19]. Regardless, the available comparative trial evidence in 1930 was thin.

The ADA CDT was among the first professional organizations to endorse vitamin D dental caries prophylaxis claims in 1930. The Medical Research Council in the United Kingdom (UK) described in 1936 how prior evidence was encouraging and promising, but insufficient to provide “trustworthy results” [20]. The UK Medical Research Council reported it was “necessary not only to extend it (i.e., the preceding evidence) to a much larger institution, but to apply the tests to the ordinary child and not simply the sick child, and also to carry on for a longer period” [20]. The AMA Council on Foods and Nutrition did not endorse a dental caries prevention claim for vitamin D until 1944.

**Key point:** The ADA CDT started endorsing vitamin D dental caries prophylaxis in 1930 when comparative trial data were in an exploratory stage. These pilot data were promising and would lead to many trials being conducted.

#### 1.1.2. The Public Health Risks of Rejecting Vitamin D Dental Caries Prophylaxis in 1944

The ADA CDT endorsed vitamin D dental caries prophylaxis until 1944 when it became committed to serving public health. On 20 July 1944, the ADA CDT had unanimously approved to change their motto from “We serve not for ourselves but for dentistry” to “To serve dentistry and promote the public welfare” p. 290 in [21]. The ADA CDT was described in JADA in 1944 as having an “unflagging fight to protect the public” [12].

The public health considerations on the role of vitamin D deficiencies in dental caries prevention were significant in 1944.

*Dental caries was rampant:* In a 1941 press conference, US President Franklin D. Roosevelt reported that over 20% of those who were called to serve in the military were rejected because of dental problems which he described as “the principal problem” which needed to be addressed “first” [22,23]. The caries problem was not limited to the military. An editor of a dental journal reported that some viewed the dental profession as failing to provide adequate care for eighty percent of the US population [24]. More than 96% of those aged 15 had dental caries [25].

*Primary prevention of dental caries had become an ADA goal:* The ADA Bureau of Public Relations released a study in 1944 showing that about 130,000 dentists were needed to take care of dental problems in the US population. Only 65,000 dentists were available [26]. Solutions to this dental manpower problem, according to this ADA Bureau report, had to include “basic research” on “how the incidence of dental disease in the whole population can be decreased” [26]. The American Public Health Association in 1944 adopted a resolution to endorse and support efforts for “research to reduce the incidence of dental disease” [27].

*Vitamin D dental caries prophylaxis was the only ADA CDT accepted primary prevention*: In 1944, the ADA CDT did not accept dental caries prevention claims for toothbrushes, toothpastes, oral rinses, or chemotherapeutics [28]. Neither did the ADA CDT accept fluoride. In 1944 the ADA CDT stated that “the routine use by the dental profession or by the laity of foods, drugs, mouth washes, dentifrices and other preparations to which fluorides have been added is not justified because of their questionable value and their known potential deleterious effects from the systemic absorption of fluorides” [29].

*A high childhood prevalence of vitamin D deficiencies:* Forty-seven percent of children (ages 2–14) born between 1927 and 1942 had rickets-a sign of severe vitamin D deficiency [30]. Accepted Dental Remedies included in its 1944 edition a citation indicating there was “abundant evidence” that the US diet was not “fully adequate” in nutrients such as vitamin D for optimal skeletal and dental mineralization. The widespread lack of use of vitamin D supplements in children was suggested as responsible for a high prevalence of childhood dental caries and osteoporosis [31].

*Dental caries informs on the human vitamin D requirement:* An article in the *Journal of the American Medical Association* (JAMA) prepared under the auspices of two AMA Councils and cited in the 1944 edition of Accepted Dental Remedies reported that “the prevention and arrest of dental caries” was the only criterion to have “a considerable degree of usefulness” for defining a vitamin D deficiency in childhood [31]. A hypothesis was emerging that the pediatric vitamin D needs for dental caries prevention had been underestimated. The AMA Council on Foods and Nutrition evaluated in 1941 whether vitamin D intake should be increased from 400 to 600 International Units (IU) [32].

**Key point:** The ADA CDT was deciding on rejecting vitamin D dental caries prophylaxis when primary dental caries prevention had become an ADA priority, when no other primary prevention approaches were available, and when two AMA councils authorized the publication of the view that pediatric vitamin D deficiencies are prevalent and causing pediatric osteoporosis and dental caries.

#### 1.1.3. An Inexplicable ADA Reversal on Vitamin D Dental Caries Prophylaxis

**Figure 1:**
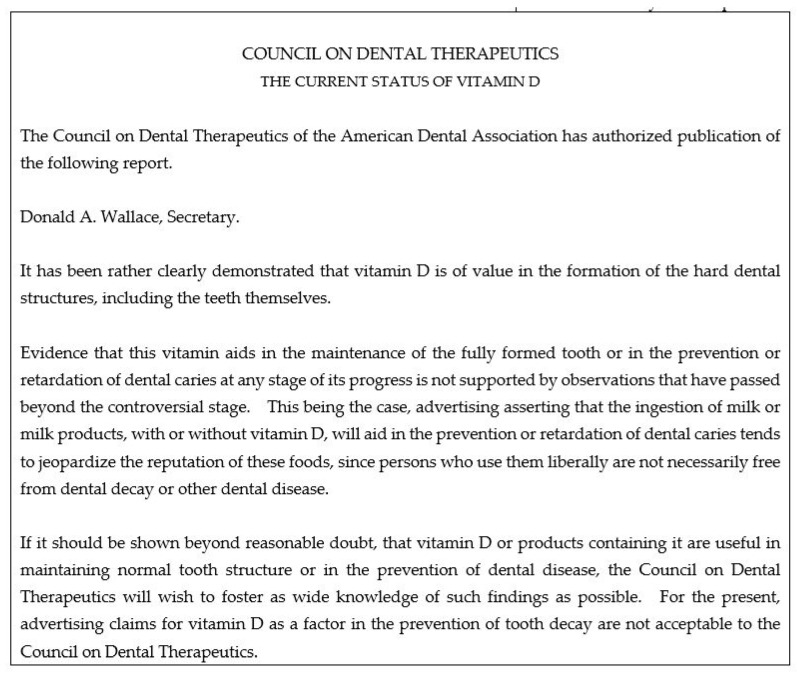
The 1945 ADA announcement that claims for vitamin D as a factor in the prevention of tooth decay are not acceptable. The second paragraph of this announcement focuses on arguments that the reputation of milk as a good source of minerals (with or without vitamin D) would be ruined if advertisers can claim that milk prevents dental caries (because people still would get cavities even though they were drinking milk). This reference to milk was another puzzling element in the ADA reversal. The ADA CDT did not endorse foods; this was within the purview of the AMA Council on Foods and Nutrition. The Journal of the American Dental Association had published in 1943 over a dozen advertisements in support of dairy products, including 2 advertisements for vitamin D milk [34,35]. Furthermore, the fact that people consuming adequate amount of vitamin D are not necessarily free from dental caries was well accepted. The “New and nonofficial remedies” publication of the AMA and “Accepted Dental Remedies” of the ADA had specified in 1936 and 1941, respectively, that there is no warrant for the claim that an adequate vitamin D intake will prevent dental caries [36,37]. This announcement can only be fully understood with the help of the ADA internal records. This ADA announcement was a reaction to the AMA vitamin D advertisement show in Figure 2.

On 29 December 1944, the Secretary of the ADA CDT had obtained the necessary votes to approve the announcement that “claims for vitamin D as a factor in the prevention of tooth decay are not acceptable” [33]. On 1 February 1945 the announcement that vitamin D played no role in dental caries prevention was published in JADA (Figure 1). The question why the vitamin D dental caries prophylaxis claim came up for debate in 1944 is addressed in the next section. This section aims to assess on which evidence the ADA CDT reversed within the context of the scientific rules they had adopted.

An unexplained ADA CDT reversal: The ADA claim that vitamin D played no role in dental caries prevention consisted of 5 sentences, presented no evidence, and had no research citations (Figure 1) [38]. The 1946 edition of Accepted Dental Remedies changed the description of vitamin D from “not the most important factor” (in 1945) to “not an important factor” (in 1946) in dental caries prevention and also provided no citation to support this revision [39]. As will be shown shortly, the ADA decision was controversial. Despite this, the ADA CDT did not invite experts to write an article explaining the reversal—a common ADA approach to deal with controversies. Also, the ADA CDT did not organize a symposium, another common approach of that era to settle controversies. 

The writing panel for the National Academy of Sciences noted this lack of evidence in 1952 and described the ADA reversal on vitamin D effectiveness as based on opinion, and void of evidence [3]. 

A controversial ADA CDT reversal: From 1944 onward, four independent writing panels evaluated the evidence on vitamin D and dental caries prevention and came to the opposite conclusion as the ADA CDT.

1. In 1944, the ADA was informed that the AMA Council on Foods and Nutrition had taken the position that “vitamin D is a beneficial factor in preventing and arresting dental caries when the intake of calcium and phosphorus calcium and phosphorus is liberal” [40].
2. In 1946, the AMA Council on Pharmacy and Chemistry endorsed vitamin D dental caries prophylaxis [41]. This AMA Council operated with roughly the same official rules as the ADA CDT, had presumably the same published evidence at its disposal as the ADA CDT, and yet reached the opposite conclusion of the ADA CDT.
3. In 1947, a Michigan Workshop on dental caries was organized and 114 “well-known research workers” were divided into 6 working groups/topics, including an 8-member vitamin and mineral group [42,43]. This group concluded that “there is evidence to suggest the possibility of increased susceptibility to caries attack in teeth which have been formed during a condition of vitamin D deficiency.”
4. In 1952, the National Research Council of the National Academy of Sciences reported that there was a “preponderance of evidence” that adequate amounts of vitamin D prevent and retard dental caries [3].

These 4 panels endorsed vitamin D during and after 1944, and therefore, whatever may have prompted the ADA CDT to reject the vitamin D dental caries prophylaxis claim, was unconvincing or unknown to these panels. The ADA CDT did not disclose what evidence prompted their reversal. And a scan of clinical studies of that era similarly fails to identify what this evidence may have been [2]. 

Four writing panels contradicted the ADA CDT and yet the ADA CDT did not offer a rebuttal [3,41,43]. Remarkably, there were three members of the ADA CDT panel who voted on the vitamin D reversal and who also took part in the 1947 Michigan Workshop [43]. These three members did not take the opportunity on the conference day specifically reserved for discussion to inform the vitamin and mineral group on what had prompted the ADA CDT to conclude the opposite.

An unjustifiable ADA reversal: Official ADA rules in 1944 indicated that “particular weight” in the assessment of dental product claims should be given to “whether recent evidence has substantiated claims.” “Recent” was defined as in the previous three years [44]. Official ADA CDT rules furthermore stated that “very strong evidence is needed when the claim is contrary to accepted scientific data”, and that “comparative trials facilitate and are often necessary for such judgment” [44]. These aspects of the scientific rules had essentially remained unchanged since 1930.

A systematic scan of the literature revealed two comparative trials published in the three years preceding 1944 [2]. Thus, there were only two comparative trials published which could provide the ADA CDT with the “very strong evidence” required for the reversal. To what extent one can assume that the ADA CDT experts (who were about to reverse on vitamin D) were aware of these two trials cannot be determined. The ADA official rules however do suggest that the ADA CDT should have been aware of at least one, and possibly both trials on two grounds. 

First, a 1943 vitamin D advertisement published in JADA cited and summarized one of these two recent trials [45]. This trial was published in 1942 in JADA (*n* = ~ 250 children) and concluded that 800 units of cod liver oil in milk reduced the number of new caries surfaces per child by 63% [46]. This JADA advertisement carried the ADA Seal which—according to ADA Seal rules—implied that the ADA CDT had reviewed and accepted the evidence. 

Second, the ADA CDT expert on vitamins added sometime after May 1944 a new citation [47] for the upcoming 1945 edition of Accepted Dental Remedies. This citation referred to the aforementioned 1942 trial and also to the second trial which was published in 1941 (*n* = ~ 200 children). This 1941 trial reported vitamin D supplementation reduced dental caries by up to 75% [48]. Theoretically, ADA CDT members other than the ADA CDT vitamin expert could have been aware of this second trial as revisions in Accepted Dental Remedies were discussed.

The comparative trial evidence published in 1941 and 1942, i.e., the recent evidence relative to 1944, in combination with the prior trials, may have justifiably led four authoritative writing panels to endorse vitamin D dental caries prophylaxis in 1944, 1946, 1947, and 1952. 

The comparative trial evidence published in 1941 and 1942 contradicts and explicitly does not justify the ADA reversal within the context of the official rules the ADA CDT had adopted. The ADA had endorsed vitamin D dental caries prophylaxis in 1930 based on two (possibly three) small trials [17,18]. The ADA continued to endorse vitamin D when US philanthropy-funded studies reported “striking” beneficial results for vitamin D and minerals in 1934 [49]. The ADA continued to endorse vitamin D when the large UK government-funded trial in 1936 reported “impressive” reductions in the initiation and spread of caries which “convincingly” confirmed the pilot studies [20]. And in 1944, after the publication of two recent trials with striking positive results, the ADA CDT reversed and claimed vitamin D played no role in dental caries prevention.

**Key point**: The ADA reversal on vitamin D was authorized by a scientific council and yet was inexplicable from the perspective of the council’s scientific rules.

### 1.2. An Inexplicable Opinion Explained?

*Manufacturers ‘desire to advertise the large-headed (tooth)brush in a journal of such prestige as the J.A.M.A, especially since the legend “Accepted for advertising in publications of the A.M.A.” appears on the ad.’*

*JAMA frequently contained advertisements for toothbrushes and their therapeutic value. Donald Wallace, Secretary of the ADA CDT, informed the ADA CDT on the unsuccessful efforts of ADA governing bodies to reach an understanding with representatives of JAMA on what therapeutic claims of dental significance could be advertised in medical journals [50]*.

#### The ADA Bulletin Informs on the Inexplicable

ADA had a complex governing structure which included a President, a Business Manager, a Board of Trustees, a House of Delegates, and a General Secretary [51]. This governing body had authority over ADA committees, commissions, bureaus, and councils, including the ADA CDT. Unlike the ADA CDT, ADA governing bodies are not necessarily bound by a set of scientific rules in their decision making [51]. 

Internal documents indicate that ADA governing bodies initiated the events which led to the ADA CDT to flip-flop on vitamin D and topical fluoride, the latter being relevant to the vitamin D story. 

**Figure 2:**
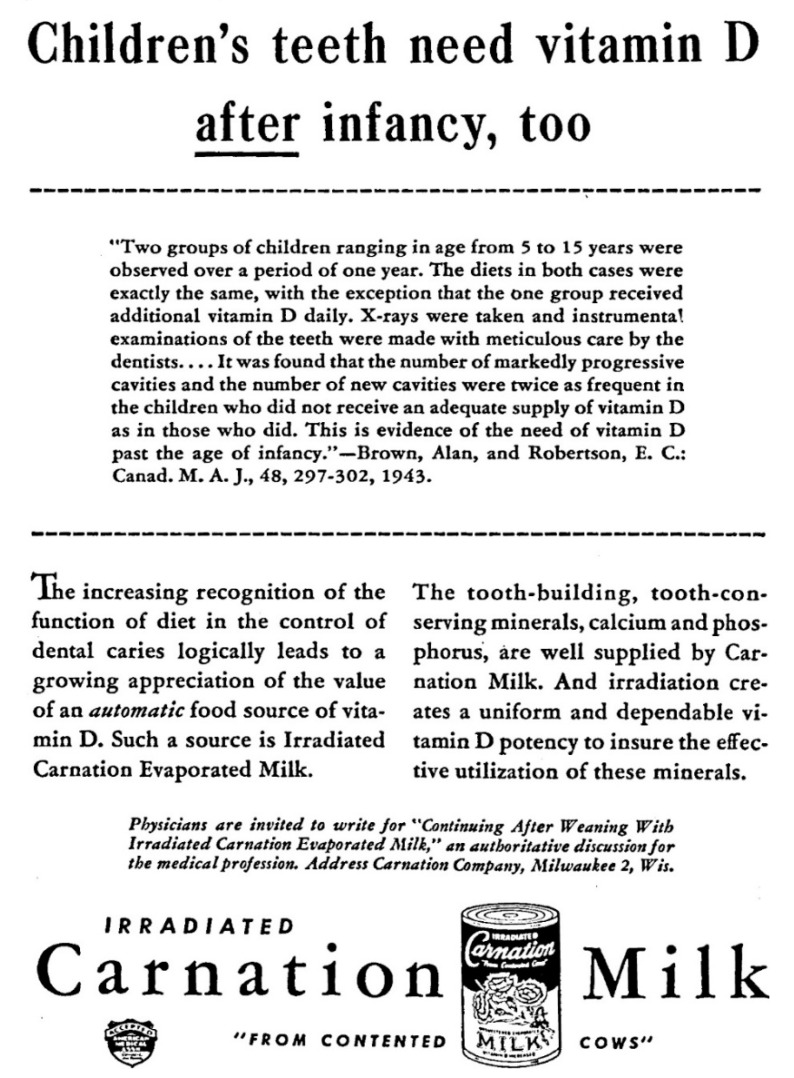
This milk advertisement was published in the Journal of the American Medical Association, endorsed by the AMA Council on Foods and made pediatric dental claims. Internal records show that it was this particular advertisement which led the ADA CDT to authorize the ghost-written opinion that “claims for vitamin D as a factor in tooth decay are not acceptable” [52]. Advertisement provided by Nestlé. Used with permission.

For vitamin D, the ADA CDT was informed that the ADA Business Manager, who is part of the governing body at the ADA, was “disturbed” by dental advertising claims in medical journals p.148 in [21]. The most cited concern was that the AMA was advertising toothbrushes in their medical journals. It is within the context of this conflict that the Secretary requested the ADA CDT to flip-flop on vitamin D—the medical profession’s approach to dental caries which was also advertised in medical journals (Figure 2) [21]. Internal documents show the ADA CDT acquiesced to the request. Internal documents confirm the ADA CDT did not abide by the scientific rules they had adopted; they did not cite or discuss recent scientific evidence which could justify their reversal [21]. It should be emphasized that the ADA CDT’s reasons for reversing on their prior endorsement are heterogenous, complex, and partly unrelated to the concerns of the ADA business manager. The ADA CDT views will be reported on separately—the key point here is to highlight that it was a conflict on advertising policies between two governing bodies of professional organizations which informs on the reversal on vitamin D dental caries prophylaxis, not the ADA CDT scientific rules. 

The ADA CDT was aware their announcement would be on the opposite side of authoritative writing panels. One ADA CDT member voted “no” on the reversal as he considered it would be “unfortunate” for the ADA CDT “to go on record as in disagreement with the Council on Foods and Nutrition of the A.M.A.” p. 467 in [21]. This same council member also expressed his concern of going opposite of the *National Academy of Sciences* as follows: “Jeans (a physician) is a member of the National Research Council which has made a printed statement, and I think we need to be a little careful how we come out in opposition to it” [53]. This concern was expressed a few months after the reversal was published.

For fluoride, the ADA CDT was informed in 1947 that the subject was discussed in “great deal in the (ADA) central office” and it had been decided to publish an editorial in JADA that topical fluoride, when “properly applied by the dentist”, was safe and effective. The ADA CDT was put in front of a fait accompli [54]. The ADA CDT decided to flip-flop on the safety of topical fluoride to fall in line with ADA governing bodies [29,55,56,57]. One involved ADA CDT member reported how this sequence of decision making was “bad policy”, how it might be perceived that their hand was forced on fluoride because if the ADA CDT were to take “a contrary stand (to ADA governing bodies) there will be an embarrassing conflict” [58]. 

The ADA General Secretary described the relationship between the ADA CDT and prior holders of his office on the day of the fluoride flip-flop as “somewhat long and stormy” [59]. His assessment confirms historical analyses of this relationship [10,60]. 

**Key point:** Members of ADA governing bodies, and not the ADA CDT, made the key 20th century decisions to initiate the reversals on both vitamin D and professionally applied topical fluorides. 

### 1.3. Globalizing an Inexplicable Opinion

JADA “*has the largest paid circulation and the widest paid distribution, of any dental publication in the world. -the journal builds dentistry*” [61].

#### 1.3.1. ADA Exerting Influence on Standards of Dental Care

ADA CDT shaped education, clinical practice, and research, often on a global scale. The ADA CDT’s work was described as recognized by the Food and Drug Administration and the Federal Trade Commission. The ADA CDT was reported as cooperating with the AMA [62]. Copies of Accepted Dental Remedies were shipped to leaders in the US Public Health Service and the US military [63]. Accepted Dental Remedies—an ADA publication—was described in 1935 as required or recommended reading at almost every US dental school [64] and would become translated into Portuguese and Spanish [64]. The ADA CDT’s work was described as being commended in almost every quarter of the globe [64]. The ADA CDT’s work was published in JADA which in 1940 was sold in 82 countries [61]. The ADA CDT’s work delineated standards of dental care. Practicing dentists who went against “the authority and prestige” of the ADA CDT were warned in journal articles and advertisements about the risk of malpractice lawsuits [62,65]. 

This stature of the ADA CDT impacted conventional wisdom—dental students after 1946 read in Accepted Dental Remedies that vitamin D was “not an important factor”; clinicians subsequently put themselves at risk of malpractice when prescribing vitamin D for dental caries prophylaxis.

ADA governing bodies can shape conventional wisdom through other direct lines of authority. The Board of Trustees for instance appointed a new JADA editor in 1947 who had been a director of the ADA Bureau of Public Relations since 1933, a branch within the ADA which promoted oral hygiene. Under his editorship, an editorial on new weapons for dental caries prevention referred to the Michigan Workshop [66] and listed tooth brushing as a practical measure against dental caries, the intervention for which the Workshop concluded there was “little scientific evidence” [43]. This editorial on the Michigan Workshop did not mention vitamin D, the intervention for which the Workshop concluded there was evidence [66] No articles with the words “vitamin D” in the title could be identified during his 15-year tenure as JADA editor.

#### 1.3.2. ADA Exerting Influence on the Dental Research Agenda

The ADA sponsored the bill to create the National Institute of Dental Research (NIDR)—an institute which a US senator described as aiming to prevent teeth from being filled. This institute would become credited with motivating 17 countries to fluoridate their water, for training hundreds of foreign dental scientists, and for funding research in 7 foreign countries. The New York Times described the first two NIDR directors as fluoride pioneers (there were 4 such recognized fluoride pioneers) [67,68]. These NIDR directors’ were ADA members and had a long prior history of research on fluorides and micro-organisms [69]. The term fluoride appears extensively in the index of a book on the 20th century history of NIDR; the term vitamin D is not in this index. Vitamin D is briefly mentioned in this historical reference work in defense of the first NIDR director’s view that dental research was “somewhat chaotic” and how “even the nutritionists disagreed amongst themselves” [70]. 

This NIDR fluoride research agenda was first put in view at US Senate Hearings on the ADA bill in 1945 when the ADA CDT’s official position still was that fluoride had “unestablished value and known potential deleterious effects”. The ADA star-witness in the US Senate was the US Surgeon General who testified that topical fluorides appeared “very promising” and that there was a suggestion that water fluoridation would reduce the amount of dental caries by one-half [71]. The Surgeon General furthermore testified “that we have no specific method of prevention of dental caries” [71]. With this statement, the US Surgeon General may have ignored the emerging views of at least 2 independent scientific councils. The AMA and the National Academy of Science both were on their way to officially endorse vitamin D dental caries prophylaxis [72,73]. The ADA CDT was likely also still endorsing vitamin D dental caries prophylaxis when the Surgeon General was preparing documents which were going to be to be inserted into the record of the US Senate Hearings [74]. The ADA CDT officially reversed its position on vitamin D just 5 months before the US Senate Hearings.

Other ADA witnesses testifying in front of the US Senate included the ADA director of Public Relations, the ADA director of the Committee on Dental Economics, the ADA chair of the Council on Dental Health, the ADA chair on the Committee of Legislation, a member of the ADA War Service Committee, the ADA president-elect, and the JADA editor. No members of the ADA CDT—the council responsible for ADA scientific affairs—testified at the hearings focused on creating a science institute.

#### 1.3.3. ADA Exerting Influence on Global Dental Public Health Policies

ADA governing bodies played a significant role in creating the dental branch at the WHO, an organization which shaped global dental public health messages. The WHO’s monthly magazine was published in French, Spanish, Portuguese, and English and illustrates how our current global conventional wisdom was shaped. In 1966, this WHO magazine educated laypeople on dental issues and described how water fluoridation in each case “yielded the same spectacular results”, how “the importance of oral hygiene cannot be underestimated”, how “dental surveys have shown again and again that …there is no direct correlation between specific nutritional deficiencies and caries.” [75]. The authors of this WHO magazine described dental caries as an infection, and rats as sometimes appearing “to enjoy” having anti-caries substances applied to their teeth [75]. The JADA editor noted that ‘through its wide distribution the message on the importance of oral health will be read throughout the world.’ [76]

The creation of a WHO message which ignored a preponderance of evidence on vitamin D started in 1946 (i.e., right after the ADA CDT’s reversal on vitamin D) [77]. An editorial in JADA written when Hillenbrand was editor reported how the ADA had to bring ‘the necessary facts to the attention of proper authorities of the World Health Organization’ [77]. An ADA resolution was passed in 1946 to have dental representatives “appointed as dental advisers to the delegates of the World Health Assembly from the various nations”. This ADA resolution was sent to a large number of leading groups and people including the President of the United States [78]. 

Hillenbrand, part of the ADA governing structure, was the first dentist to serve as an adviser to the United States delegation to World Health Organization [79]. He had “on his own responsibility” in 1947 decided to endorse topical fluorides by dentists when the official ADA CDT position was still to reject such an endorsement in part because “the full extent of their possible harmful effects are not known” [55]. 

Knutson, a lifelong ADA member and fluoride pioneer, followed up in the creation of a dental branch at the WHO. He advocated fluoride [80] and belittled nutrition by quoting Pliny (23/24 to 79 BCE): “If one wishes to be free of toothache, one should eat a whole mouse twice a month” [25]. Knutson was present as a dental officer of the US Public Health Service at the Senate Hearings on the creation of NIDR in 1945. In 1954-1955 he chaired an international group to establish and organize a permanent WHO dental health program. During these two years he spent over 6 months in Geneva [81,82]. In 1956, he became a member of the ADA governing body (elected as the 3rd ADA vice-president). In 1958, he co-authored the technical report of the WHO [83] on water fluoridation which described vitamins as disappointing in dental caries prevention. Ericsson and Hodge were two co-authors on this WHO report. The first co-author, Ericsson, had been a research fellow with Knutson at the US Public Health Service in 1952 [84]. The second co-author, Hodge, was the ADA go-to-scientist on fluoride issues who submitted a letter to the US Senate Hearings in support of creating NIDR and who received an honorary ADA membership [85]. The technical report was described in JADA as “the WHO … approving water fluoridation” [86]. 

#### 1.3.4. ADA Exerting Influence on Global Professional Standards

The ADA played a role in the re-start of the FDI where by 1963 FDI fluoridation advocates reported how fluoridation plants were in operation or “will start in the near future in 41 countries.” [87] The FDI’s agenda may also have been influenced by the ADA political leadership. The ADA International Relations Committee reported in 1943 how the ADA “has a responsibility in seeing that American dental information is widely disseminated to other countries” [88] and in 1946 this committee helped with renewing scientific and fraternal ties with Europe and the rebirth of the FDI [89]. Knutson, the fluoride pioneer, was in 1956 active at the FDI as the vice-president of the Commission on Public Dental Health Services and chairman of the FDI Committee on Statistics [82]. Another fluoride pioneer and NIDR director, Arnold, was Vice President of the Scientific Committee at the FDI from 1954 until 1961 [67,90]. Ericsson, an author of the WHO report on fluoride and past colleague of Knutson was an FDI member from 1958–1966 [84]. 

**Key point**: ADA governing bodies had several channels of influence to put fluoride experts on authoritative writing panels who globalized the now conventional wisdom of ignoring and dismissing the evidence on the role of nutritional deficiencies in dental disease etiology.

### 1.4. A Preponderance of Clinical Trial Evidence Becomes Heresy

*A patient with a vitamin deficiency “should be referred to the physician who …does have the training and the facilities for a general physical examination of the patient which the dentist can’t claim to be trained for or have the equipment to do it.”*

*Milan Logan, a biochemist, and lead vitamin expert of the ADA CDT reflecting on his unpopular opinion that the treatment of nutritional deficiencies fell into the medical scope of practice* [91].

Unlike the ADA, the AMA did not announce that vitamin D was ineffective for dental caries prevention. Instead, in 1958 the endorsement of vitamin D dental caries prophylaxis disappeared from the AMA New and Nonofficial Drugs, a yearly publication [92]. The evidence had not changed; what changed was that dentistry was separating from medicine [93]. The dental profession largely won a scope-of-practice conflict with the medical profession; physicians largely stopped learning about dental diseases in medical school, the medical profession stopped endorsing toothbrush advertisements in their medical journals. Research into the medical management of dental diseases, including the role of nutritional deficiencies in dental disease prevention, became abandoned by the medical profession because of this separation. 

The ADA reversal on vitamin D dental caries prophylaxis may have been the most challenging step in terms of dentistry separating from medicine—vitamin D was the crown jewel of the medical management of dental diseases, studied for over 25 years, and the only medical management approach of dental caries which was endorsed by the dental profession. The ADA reversal on vitamin D, as was shown here, was controversial but nonetheless became the conventional wisdom. Dismissing the role of other nutritional deficiencies in the etiology of dental diseases was less challenging.

A vitamin C deficiency offers an informative example of how another nutritional deficiency with dental symptoms became ignored. The AMA Council on Foods and Nutrition listed gingival bleeding as a potential sign of a subacute vitamin C deficiency in 1946 [94]. The National Academy of Medicine described an increased gingival bleeding tendency as one of the most sensitive markers for a vitamin C deficiency in 2000 [95]. Clinical trials focusing on bleeding tendency confirmed these conclusions [96]. Nevertheless, the conventional wisdom is to largely ignore a vitamin C deficiency in the etiology of gingival bleeding. Instead, a non-controlled small case-series on experimental gingivitis, a study cited in dental journals over 2,100 times, became the bedrock citation to justify treating gingival bleeding with oral hygiene, an approach which assuredly fails at correcting a vitamin C deficiency [97,98,99]. 

Research into the role of other nutritional deficiencies, which was often conducted by physicians, such as calcium, phosphate, vitamin B6, and vitamin K became abandoned in a similar fashion [3,100,101]

The National Academy of Sciences concluded in 1989 that the “paucity of more recent evidence” suggested that vitamin D did not play a major role in dental caries prevention [5]. The “paucity of more recent evidence” may have occurred because research questions and funding had become re-framed to questions which fell into the dental scope of practice, not the medical scope of practice. As reported by Brownell and Warner, “A great deal of influence rests in the hands of parties who control the framing of a health issue.” [102]. The dental profession going forward largely framed research questions in such a way as to exclude the medical management of dental diseases. 

**Key point:** The medical and dental profession separated leading to an abandonment of the medical management of dental diseases.

## 2. Discussion

ADA interviewer: “*…the ADA shall represent both the interests of its members and the public that its members serve. Is this truly possible?*” [103]

Harold Hillenbrand, ADA Chief Executive officer (1947–1970): *“yes, it is possible to combine the two goals as stated*…”

This historical analysis indicates that the American Dental Association endorsed vitamin D dental caries prophylaxis for 15 years, and then reversed in a controversial fashion—4 authoritative writing panels disagreed with the ADA CDT. Other professional societies have found themselves similarly at the center of controversial reversals. The American Heart Association first reported that dietary fats were not associated with coronary heart disease; and then, reversed itself to the opposite position in 1961 [104]. The American Diabetes Association reported that diabetics should avoid simple sugars; and then, reversed itself to the opposite position in 1994 [105]. World-leading professional societies can then create an anchoring bias and generations of health professionals can become educated and trained as if these controversial reversals are supported by unequivocal evidence. 

Reversals offer a unique opportunity to scrutinize what motivates professional organizations to flip-flop on the scientific data which they had previously accepted. This historical analysis focused on the ADA reversal on vitamin D as a dental caries prophylactic. The ADA internal documents showed that the reversal announcement found its origins within the context of conflicts on advertising policies. The American Heart Association and the American Diabetes Association justified their reversal with what has now become described as fragile evidence [106,107,108]. Possibly, hidden conflicts similarly motivated these latter 2 professional organizations to make pivotal decisions on fragile science.

These professional organizations of clinical specialists can translate opinion or fragile evidence into bold public health policies [109] by presenting its views on the “necessary facts” to other organizations. A 190-word opinion statement by the ADA with no scientific references is the basis for our current conventional wisdom on treating nutritional deficiencies with fluoride. A 7500+ word analysis by the National Academy of Sciences with over 45 scientific references, and with opposite conclusions of the ADA, became almost regarded as heresy. Current conventional wisdom could have been different if the National Academy of Sciences was provided with the political power to present its “necessary facts” to the dental profession, to the NIDR, to the WHO, and to the FDI. Even in 1952, the writing panel for the National Academy of Sciences still expressed some skepticism on water fluoridation reporting that it remained to be proven that “fluoride added to a soft water had the same action as natural fluoride-containing water” [3]

The power of professional organizations of clinical specialists to shape conventional wisdoms despite a slew of red flags is remarkable. It did not matter in this case that the specialists created a conventional wisdom which was opposite of a preponderance of evidence. It did not matter that the professional organization had a self-evident conflict of interest; topical fluoride applications in dental offices were revenue-generating procedures, vitamin D prescriptions were not. It did not matter that the National Academy of Sciences and the AMA came to opposite conclusions of a specialist professional organization. None of these red flags mattered—clinical specialists were considered trustworthy even when controlled trial evidence suggested that their expert opinions could be causing harm. That the WHO and other organizations ignored these red flags and blindly adopted the opinion of conflicted specialists is remarkable. 

The main strength of this report was to provide an evidence-based assessment of the decision process making of a scientific council within the context of the rules they were operating under. This report was thus not an anachronistic interpretation of scientific events. This report has several weaknesses. The history was analyzed from the perspective of the ADA—it ignored analyzing the process from the AMA perspective. The AMA also did not cite evidence why they started endorsing a vitamin D dental caries prophylaxis claim just when the ADA announced they would stop endorsing such a claim [3]. An examination of the conflict from the perspective of the AMA side would show that both organizations were driven by one and the same conflict on advertising policies, and further research needs to be determine whether the AMA was equally compromised on adhering to their scientific rules (on therapeutic claims for toothbrushes) in this inter-professional conflict. One ADA CDT member made up his opinion on this question in the middle of the conflict: “Obviously, (the JAMA editor) has little but financial interest in dental advertising in the J.A.M.A” [110]. Independent of conflicts on advertising policies, an examination of the conflict from the perspective of the AMA shows one powerful figure in opposition to the ADA CDT-Jeans, a pediatrician, a vitamin D researcher, and a prominent AMA figure. Two, possibly three, of the writing panels coming out in opposition to the ADA may have been influenced by Jeans and may thus not have reflected independent scientific interpretations of the evidence.

Other weaknesses of this report include that within-ADA conflicts were ignored; the aim was to focus on the origins of our current conventional wisdom—the dominant view. It needs to be re-emphasized that the actual views of individual members of the ADA CDT on the reversal remained undiscussed here and will be reviewed separately. Neither does this report imply that the non-dominant view necessarily became heresy in all quarters after the ADA reversal. The NIDR for instance did fund a study on vitamin B6 (pyridoxine) and dental caries [100]. Other weaknesses include the lack of analysis on how other ADA bureaus, councils, and committees shaped policies, and how oral hygiene and pharmaceutical industries influenced both conventional wisdom and professional organizations such as the ADA or the AMA. 

In summary, this historical analysis further substantiates the recommendation by the National Academy of Medicine that clinical specialists are not necessarily trustworthy when it comes to writing clinical guidelines [111]. In the end, some members of the ADA scientific council bear responsibility for authorizing a reversal on vitamin D dental caries prophylaxis within the context of a conflict on advertising policies. This historical analysis also shows that scientific councils are but a small cog in professional organizations, a cog which can be coaxed and replaced by governing bodies. Public health may well depend on looking at professional societies no different than the way we look at the pharmaceutical industry—conflicted organizations with a power to shape conventional wisdom based on fragile evidence. This historical analysis adds to the evidence that professional societies should serve their members and be kept at arm’s length from research agendas, disease definitions, clinical practice guidelines, and public health policies.
